# An In Vitro Study of the Number of Distal Roots and Canals in mandibular First Molars in Iranian Population

**Published:** 2008-01-10

**Authors:** Hasan Razmi, Noushin Shokouhinejad, Mohsen Hooshyar

**Affiliations:** 1 *Department of Endodontics, School of Dentistry/ Dental Research Center, Tehran University of Medical Sciences, Tehran, Iran and Iranian Center for Endodontic Research*; 2 *Department of Endodontics, School of Dentistry/ Dental Research Center, Tehran University of Medical Sciences, Tehran, Iran*; 3 *General Practitioner, Tehran, Iran*

**Keywords:** Anatomy, Molar, Population, Root Canal

## Abstract

**INTRODUCTION:** The purpose of this study was to evaluate the number of distal roots and canals in mandibular first molars and their internal anatomy radiographically within Iranian population.

**MATERIALS AND METHODS:** A total of 310 distal roots of mandibular first molars were incorporated in this study and evaluated in terms of number of roots and number and types of canals. Root canal systems were studied in vitro by means of radiography and based on Vertucci’s classifications.

**RESULTS:** It was shown that 4.5% of the teeth in this study had two distal roots, of which, 100% indicated type I for both distobuccal and distolingual roots. Among all the teeth, 43.2% had two canals, 24.2% two apical foramina, and 38.7% two orifices in their distal roots. According to Vertucci’s classification 54.9% of the teeth were type I, 19% type II, 1.9% type III, 14.2% type IV, 4.2% type V, 1% type VI, 0.3% type VII and 0% type VIII.

**CONCLUSION:** In as many as 43.2% of all teeth assessed in this study, bicanaled distal roots were observed, dentists are always recommended to search for the second canal in distal roots of mandibular first molars. In case the second canal in the distal root is missed, failure of endodontic treatment will be anticipated. A rectangular type access cavity design allows better visualization and negotiation of the probable second canals within the distal roots of mandibular first molars.

## INTRODUCTION

Obviously, the main purpose of dental services is providing people with dental and oral health and its maintenance. Knowledge of internal dental anatomy plays an important role in success of endodontic treatment and negligence of this important issue leads to improper diagnosis and treatment planning. The purpose of an endodontic treatment is chemomechanical cleaning of the root canal system, maintaining its original anatomy and obturation of this space with an inert material. In order to obtain a successful endodontic treatment, dentists must be aware of the variations in root canal morphology.

According to both in vivo and in vitro studies performed on morphology of the mandibular first molars' root canal system, it has been shown that the presence of four root canals in mandibular first molars has been pointed out in most of the credited endodontic textbooks (1), but these reports are based on studies designed in European and American populations. According to Walker (2) the need for more studies in order to gain more information about the root canals and their morphology in different ethnic groups highly exists.

Clearly, several studies have been done on internal anatomy in different countries, but since ethnic characteristics may influence internal anatomy e.g., more prevalent mandibular first molars with an additional distal root in Asians (3), more extensive research on internal anatomy of Iranian population seem necessary. Authors have shown that internal anatomy of mandibular first molars often needs more concern because the number of roots and canals in these teeth are quite variable. On the other hand, since mandibular first molar plays an important role in mastication, knowledge of the internal anatomy of this tooth is necessary to perform a successful treatment (4). According to Skidmore (5), the mandibular first molar seems to be the tooth that most often requires endodontic treatment.

**Figure 1:**
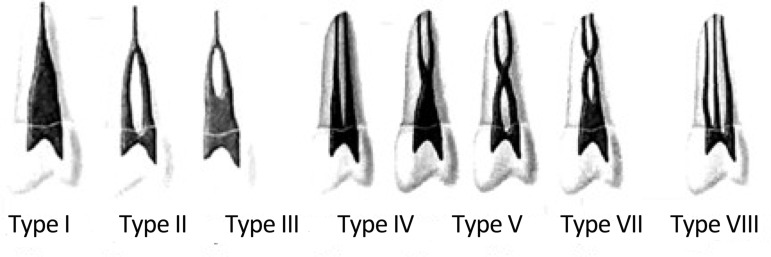
Diagrammatic presentation of Vertucci's canal configurations

The purpose of this study was radiographic evaluation of the number of distal roots, root canals and internal anatomy of the mandibular first molar teeth within Iranian population.

## MATERIALS AND METHODS

In this descriptive in vitro study, a total of 310 permanent mandibular first molar teeth with intact roots were selected. These teeth were devoid of caries and/or fracture and were not endodontically treated. The age, gender and the reason for extraction were not recorded. By the time total samples were collected, all extracted teeth were rinsed and placed in 10% formalin.

Then deposits, attached bones and soft tissues were removed by hand instruments and cleaned using a sonic device. After a thorough irrigation with tap water, the samples were placed and coded from 1 through 310 within plastic boxes containing normal saline.

The teeth were split into mesial and distal halves by means of a diamond disc. Then the distal roots were mounted in wax and radiographs were taken from the buccolingual and mesiodistal aspects of the roots (Kodak, Ektaspeed, Eastman Kodak, NY, USA) and were exposed for 0.4s using an X-ray machine set at 70kV and 8mA. The tube-to-film distance was set at 2 cm. After all radiographs were processed, the teeth codes were recorded and the radiographs were mounted in coded frames. The radiographs were assessed by an endodontist, using an X-ray viewer and magnifying lens (×2) in a dark room. Root canal systems were evaluated and classified using Vertucci’s classification (6), (Figure 1) and variability of the distal roots of mandibular first molars were evaluated in terms of number of roots, number of canals and types of canals.

## RESULTS

The results showed as follows:

**1- **95.5% of the teeth in this study had one and 4.5% two distal roots.

**2- **56.8% of the teeth had one and 43.2% two canals within their distal roots.

**3- **75.8% of the teeth had one and 24.2% two apical foramina within their distal roots.

**4- **61.3% of the teeth had one and 38.7% two orifices within their distal roots.

**5- **According to Vertucci’s classification 54.9% of the distal roots were type I, 19% type II, 1.9% type III, 14.2% type IV, 4.2% type V, 1% type VI, 0.3% type VII and 0% type VIII and in teeth with two distal roots, 100% of distobuccal and distolingual roots were type I which comprised of 4.5% of all teeth.

## DISCUSSION

In the current study, the distal roots of 310 mandibular first molars were examined radio- graphically. After evaluating the radiographs from buccolingual and mesiodistal aspects, the data were recorded according to Vertucci’s classification. However if contrast media were injected into the root canal system, internal anatomy of distal roots would be better visualized. In this in vitro study, staining- sectioning method was performed after the radiographic assessment (7) and the achieved results showed that 4.5% of the teeth had two distal roots which correspond to the radiographic assessment. Among all, 37.7% of the distal roots had two canals which are somehow less than current values. According to staining-sectioning method type of canals based on Vertucci’s classification were indicated as follows: 57.5% type I, 15.2% type II, 4.8% type III, 2.6% type IV, 4.5% type V, 0.6% type VI, 0.3% type VII and 0% type VIII. The reported values in type I, III and V were more and in types II, IV and VI less than radiographic evaluation. The values recorded for both type VII and VIII were equally presented in both studies. In teeth with two distal roots both studies showed that the distobuccal and distolingual roots were 100% type I. The reason for variability in result of different studies can be attributed to technical differences used. Staining-sectioning method can give rise to more exact results because the operator can have a direct vision and a straight accessibility to the internal anatomy of the teeth but when using radiographs the operator can be mislead by the two-dimensional image of the radiograph to assess the three-dimensional statues of the tooth. One of the limitations of current study was that only one examiner interpreted the radiographs. It is better to evaluate radiographs by two or more examiners, especially in studies that radiopaque contrast media are not injected into the root canal system.

It can be concluded from the data analysis of the two methods of staining-sectioning and radiographic assessment that both methods agreed upon number of roots, number of canals and types of canals based on Vertucci’s classification in 100%, 87.4% and 80.3%, respectively.

In mandibular first molars, additional roots can be found lingual to the main distal root and is considered more as an ethnic characteristic than a developmental anatomy. This additional root can be small-conical to fully developed shape in nature, but one must keep in mind that it contains pulpal tissue in all cases (8).

It can be inferred from the endodontic literature review concerning the frequency of mandibular first molars with two distal roots in different ethnic groups that this frequency ranges from 2.2% according to Skidmore and Bjorndal (5) to 21.5% as stated by Yew and Chan (9). The majority of the studies point at Asian ethnicity as having the highest frequency compared with other European groups, for instance in Asian groups the highest frequency was 21.5% as stated by Yew and Chan (9) and 17.8% according to de-Souza et al. (10). In European groups the highest frequency values were 3.4% based on Curzon's study (11).

Within current study, the frequency of mandibular first molars with two distal roots was reported as 4.5%. Herein, as an Asian ethnicity lower frequency are recorded compared with other Asian samples and therefore closer to European groups. This difference can be attributed to ethnic variabilities or the technique involved. Within the most Asian groups having higher frequencies, Mongols are said to be in charge of the difference (2,3,5,7,9-15), (Table 1).

**Table 1:** Comparison of the results of this study with other studies concerning the possibility of mandibular first molars with two distal roots

**Authors**	**Subject population**	**Sample size**	**Frequency of teeth with two distal roots**
Somogoyi & Simons (12)	Canadian Indian	250	16%
de Souza-Freitas (10)	European	442	3.2%
de Souza-Freitas (10)	Japanese	233	17.8%
Skidmore & Bjorndal (5)	Caucasian	45	2.2%
Curzon (11)	British	377	3.4%
Hochstetter (13)	South Korean	400	13%
Walker & Quackenbush (14)	Hong Kong Chinese	213	14.6%
Walker (2)	Hong Kong Chinese	100	15%
Yew & Chan (9)	Chinese	832	21.5%
Wasti (3)	Pakistani	30	2.3%
Gulabivala (15)	Thai	118	12.7%
Razmi (7)	Iranian	310	4.5%
Current study	Iranian	310	4.5%

It can be concluded from other investigations that the frequency of bicanaled distal roots of mandibular molars can range from 27% according to Pineda and Kattler (16) to 50% based on Wasti’s study (3). The result of any of the studies varies with each other due to the techniques used, sample size and the subject population.

Comparing the results of other studies it can be understood that there is a considerable difference between Asian and European groups. For instance, within Asian groups, the frequency of bicanaled distal root of mandibular first molars vary ranging from 50% according to Wasti (3), to 80.5% according to Gulabivala (15), where as within European groups, this value ranges from 27% according to Pineda and Kuttler (16) to 80% according to Vertucci (6).

In studies done on Asian groups, the frequency of mandibular first molars with bicanaled distal roots recorded higher percentages than others done on European groups. This difference can be attributable to both ethnic variabilities for example higher possibility of mandibular first molars with extra roots in Asians which can justify higher possibility of mandibular first molars with bicanaled distal roots.

In the present study the frequency of mandibular first molars with bicanaled distal roots is reported as 43.2% which nearly corresponds with the results mentioned for Asian populations rather than Europeans. This difference and similarity with other studies is due to the aforementioned ethnic variations (2,3,5-7,9,15-18), (Table 2). Considering the results presented by authors concerning the frequency of different morphologic types of the distal roots of mandibular first molars, it can be understood that Vertucci's types I, II and IV have the highest frequencies, discerningly (Table 3). Within the current study, the frequency of Vertucci's types I, II and IV canal configuration was reported as 54.9%, 19% and 14.2%, respectively which is in accordance with other studies. In addition, Vertucci's types III, V, VI and VII showed lower frequencies in this study. No type VIII was seen herein, matching the results gained by other studies.

In studies separately evaluating distobuccal and distolingual roots, one can understand that type I configuration has the highest frequency.

**Table 2:** Comparison of the results of this study with other studies concerning the frequency of mandibular first molars with bicanaled distal roots

**Authors**	**Type of Study**	**Sample size**	**Frequency of bicanaled distal roots**
Skidmore & Bjorndal (5)	Polyester casting resin of extracted teeth (in vitro)	45	28.9
Pineda & Kuttler (16)	Radiographic evaluation of extracted teeth (in vitro)	300	27.0
Hartwell & Bellizi (17)	Radiographic evaluation of endodontically treated teeth (in vivo)	846	35.1
Vertucci (6)	Clearing of extracted teeth	100	30
Fabra-Campos (18)	Radiographic evaluation of endodontically treated teeth (in vivo)	145	49.66
Walker (2)	Radiographic and visual (clearing) examination of teeth (in vitro)	100	45
Yew & Chan (9)	Radiographic and visual (clearing) examination of teeth (in vitro)	832	31.5
Wasti (3)	Clearing of extracted teeth (in vitro)	30	50
Gulabivala (15)	Clearing of extracted teeth (in vitro)	118	30.5
Razmi (7)	Staining-sectioning of extracted teeth (iv vitro)	310	37.7
Current study	Radiographic evaluation of extracted teeth (in vitro)	310	43.2

**Table 3:** Comparison of the results of this study with other studies concerning the configuration of the distal roots of mandibular first molars based on Vertucci's classification

**A** **u** **t** **h** **o** **r** **s**	**Sa** **m** **p** **l** **e si** **ze**	**Configuration of the distal roots of mandibular first molars based on Vertucci's classification (%)**							
		**I**	**II**	**III**	**IV**	**V**	**V** **I**	**V** **I** **I**	**V** **I** **II**
Skidmore & Bjorndal (5)	45	71.1	17.7	-	11.2	-	-	-	-
Pineda & Kuttler (16)	300	73	12.7	-	3.7	8.6	2	-	-
Vertucci (6)	100	70	15	-	5	8	2	-	-
Fabra-Campos (18)	145	52.4	24.8	-	20	2.8	-	-	-
Walker (2)	100	55	32.4	-	12.6	-	-	-	-
Wasti (3)	30	30	26.7	-	20	20	3.3	-	-
Gulabivala (15)	118	67.9	4.8	3.9	16.5	2.9	-	-	1.9
Razmi (7)	310	57.5	15.2	4.8	12.6	4.5	0.6	0.3	-
Current study	310	54.9	19	19	14.2	4.2	1	0.3	-

## CONCLUSION

Among all teeth incorporated in this study4.5% has two distal roots and 43.2% had distal roots with two canals.

The dentist must always search for the second canal in the distal root. If one is missed, it can result in failure of endodontic treatment. The rectangular form of the access cavity will lead the practitioner to a better visualization and identification of the probable second canal of the distal root.
